# The Legacy of Thomas Hodgkin Is Still Relevant 150 Years After His Death. Nothing of Humanity Was Foreign to Him

**DOI:** 10.5041/RMMJ.10284

**Published:** 2017-01-30

**Authors:** Eldad J. Dann

**Affiliations:** 1 Department of Hematology and Bone Marrow Transplantation, Rambam Health Care Campus, Haifa, Israel; 2 Blood Bank and Transfusion Service, Rambam Health Care Campus, Haifa, Israel; 3 Bruce Rappaport Faculty of Medicine, Technion–Israel Institute of Technology, Haifa, Israel

**Keywords:** Thomas Hodgkin, Hodgkin disease, philanthropy, protection of human rights

## Abstract

Current leading figures in medical science usually focus on very specific topics and use cutting-edge technologies to broaden our knowledge in the field. The working environment of the nineteenth century was very different. Medical giants of that time such as Rudolph Virchow and Thomas Hodgkin had a wide-ranging scope of research and humanitarian interests and made enormous contributions to a variety of core areas of medicine and the well-being of mankind. The year 2016 marked the 150th anniversary of the death of Dr Thomas Hodgkin. Even a brief review of his life and work proves the current relevance of the outstanding deeds of this exceptional physician, medical educator, and defender of human rights for the poor and underprivileged; his vision was far ahead of his time.

April 4, 2016 marked the 150th anniversary of the death of Dr Thomas Hodgkin (1798–1866) (Figure 1). Three biographies^1^–^3^ and many articles^4^–^7^ have been written about Thomas Hodgkin.

**Figure 1:**
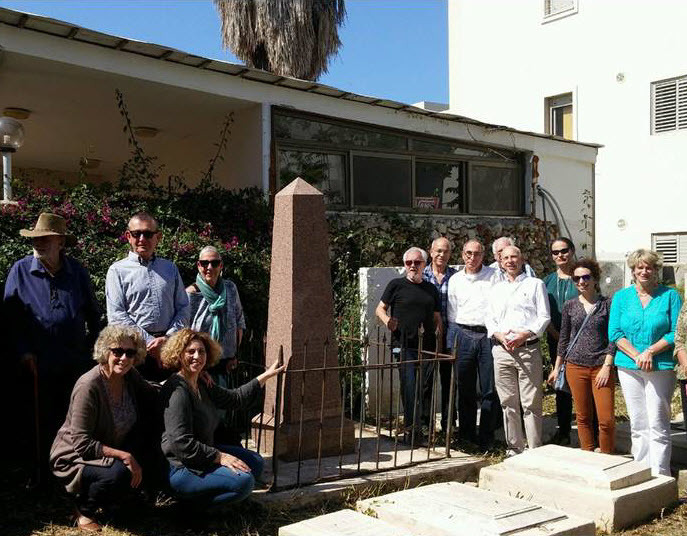
The Grave of Dr Thomas Hodgkin (1798–1866). April 4, 2016, the 150th anniversary of Dr Hodgkin’s death. Photo by E. Dagoul.

This article aims to elaborate on the features and deeds of Thomas Hodgkin that continue to make him a source of admiration and inspiration nowadays.

Thomas Hodgkin was born in Pentonville, North London, in a pious family belonging to the Society of Friends (Quakers). He was educated by his father, a teacher in the community. Hodgkin’s first workplace was with the local apothecary, starting as an apprentice under the mentorship of William Allen (1770–1843). While working there, Hodgkin was introduced to the three areas that later became his main life interests: the anti-slavery campaign that Allen had been involved in since 1807; medicine—Allen had been lecturing chemistry at Guy’s Hospital, London, for 24 years; and philanthropy—Allen had been operating a soup kitchen for the poor of Spitalfields and had been active in promoting education for the poor and underprivileged.

From his early adulthood, Thomas Hodgkin was passionate about the protection of human rights in general and of indigenous societies in particular. At the age of 21, he composed an essay, “On the Promotion of Civilization.” In this plea on behalf of endangered North American Indians he wrote: “A comparison between ancient and modern times as far as relates to the influence which civilized nations have had upon the uncivilized shows that the last five hundred years, those under the name of Christians have done far more to degrade, corrupt and exterminate their uncivilized fellow creatures than all the heathen world, since the creation of man. Wherever they have gone they have introduced new vices and new diseases.” Hodgkin offered to pay for the education of a young North American Indian, aiming to provide this person with tools for leadership in his community and for argumentative opposition to the British authorities.

Thomas Hodgkin designed scientific approaches for collecting data on endangered societies. In 1833, Dr Richard King, a renowned surgeon and naturalist of his time, took part in the expedition to the shores of the Arctic Ocean under the command of Captain G. Back of the Royal Navy. Hodgkin wrote to Dr King asking him to provide first-hand data from numerous observations and independent sources, thus ensuring the accuracy of reports and avoiding biased secondary sources, like traders and missionaries. Hodgkin founded the Aborigine Protection Society in 1837 and played a major role in the foundation of the Ethnological Society in collaboration with James Cowles Prichard. Both of them were on the committee that composed a “Manual of Ethnological Inquiry” in 1843. Hodgkin was also an advisor to the Buxton’s Select Parliamentary Committee on Aborigines’ Protection and authored several articles and a book on these issues (e.g. “A letter on Negro emancipation and American colonization,” “An inquiry into the merits of American colonization society,” and “On British African colonization society”).

In 1863, Thomas Hodgkin undertook a humanitarian mission to North Africa under the auspices of his lifelong friend, Sir Moses Montefiore. These two extraordinary persons—one a devoted member of the Society of Friends (Quakers), the other an observant Sephardic Jew—shared a wide range of humanitarian interests. Hodgkin presented an overview on the physical geography of North Africa to the Royal Geographical Society and included these materials in the book *Narrative of a Journey to Morocco in 1863 and 1864*.^8^ The book, which came out after Hodgkin’s death, was dedicated to Sir Moses Montefiore.

The contribution of Thomas Hodgkin to medicine was enormous. He started medical studies at Guy’s Hospital. After walking the ward for 2 years, he was admitted to the University of Edinburgh. In 1822, he went to France for a year of elective studies. He was privileged to work with Professor René Laennec at patients’ bedside and in the autopsy room, where he concentrated on the evaluation of clinical and pathological correlations. On his way back from France to Edinburgh, Hodgkin renewed his relationship with Guy’s Hospital. He was invited to give a lecture to the Hospital Physical Society on “mediate auscultation,” or the use of “Laennec’s cylinder,” commonly called “stethoscope,” as the means of ascertaining the changes that disease produced in the organs of respiration and circulation (Figure 2). It was the first introduction of the stethoscope to the British academic medicine; and this was done by a 24-year-old medical student! Hodgkin described the clinical pathological findings in bronchiectasis, tuberculosis, valvular diseases, and the use of auscultation during pregnancy for localizing the fetus.

**Figure 2:**
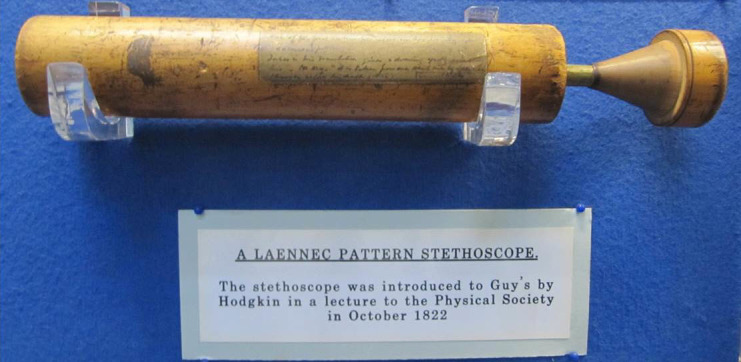
The Laennec Cylinder Used by Thomas Hodgkin. The Laennec cylinder, commonly called “stethoscope,” was introduced by Thomas Hodgkin to Guy’s Hospital. This stethoscope, used by Thomas Hodgkin, is exhibited at Gordon Museum, King’s College, London, Guy’s Campus, Hodgkin House. Photo by E.J. Dann.

From the first steps in his career, Thomas Hodgkin devoted a great part of his time to taking care of the poor. In 1825, two years after his graduation from the University of Edinburgh Medical School, he was appointed to the position of physician at the London Dispensary (a facility dealing with medical problems of underprivileged). At the same time, as a favor to Sir Moses Montefiore, Hodgkin served (until 1831) as a physician at the Society of the United Israelites and the Society of United Sisters—two Jewish philanthropic organizations. Based on this experience and in an effort to improve the medical care of this population, Hodgkin wrote an essay, “On the Mode of Selecting and Remunerating Medical Men for Professional Attendance of the Poor.”

Realizing that education for healthy behavior and abstinence from smoking was an effective way of maintaining good health, Thomas Hodgkin delivered (in 1829) a series of four lectures to laymen at the Mechanics’ Institute, Spitalfields, which he further published under the title “Lectures on the Means of Promoting and Preserving Health.”^9^ He pointed out the importance of clean air, bathing, and proper disposal of sewage. Hodgkin also warned of the dangers of overeating, excessive alcohol use, occupational dust exposure, and tobacco use, including passive smoking, stating that “smoking encroaches on the freedom and comfort of others.” His prescience was amazing, given that the first sign prohibiting smoking in Guy’s Hospital was put up in 1881, 15 years after Hodgkin’s death.

Between 1825 and 1828, while serving as the curator of the Anatomical Museum at Guy’s Hospital, Thomas Hodgkin expanded the museum collection from 500 to 3,000 specimens. Each specimen was accompanied with a label, containing information on clinical symptoms and physical signs, a reference to the inspection book where the full case and post-mortem inspection were recorded, and the name of the presenting physician. Despite such an enormous contribution, he was actually deprived of the right to use the collected materials after he left Guy’s Hospital.

Thomas Hodgkin was the first to describe many clinical and pathological correlations. In his work “On the Retroversion of the Valves of the Aorta,” published in the *London Medical Gazette* in 1829, he described aortic valve incompetence. He also reported the consequence of acute appendicitis that led to ruptured appendix, causing peritonitis and death of a young medical student at Guy’s Hospital. Hodgkin characterized differences between benign cysts and adenocarcinoma and reported a case of recurrence of uterine carcinoma a year after extirpation of the uterus, with local advancement of the tumor that led to intestinal obstruction. This case was published by James Blundell, a surgeon and a gynecologist who is remembered for the introduction of blood transfusion into clinical medicine. In this publication, Dr Blundell warmly acknowledged that “Dr. Hodgkin’s talents and great accuracy were well known to the profession.”^10^

In 1832, Thomas Hodgkin published a paper first describing the clinical entity of the absorbent gland and spleen in six patients (Figure 3). Dr Samuel Wilks, who included some of Hodgkin’s patients in his own series of case reports, suggested in his review, published in 1865, naming this disease after Hodgkin. In 1926, Herbert Fox confirmed microscopically that three of the six patients originally reported by Hodgkin actually did have “Hodgkin’s disease.”

**Figure 3:**
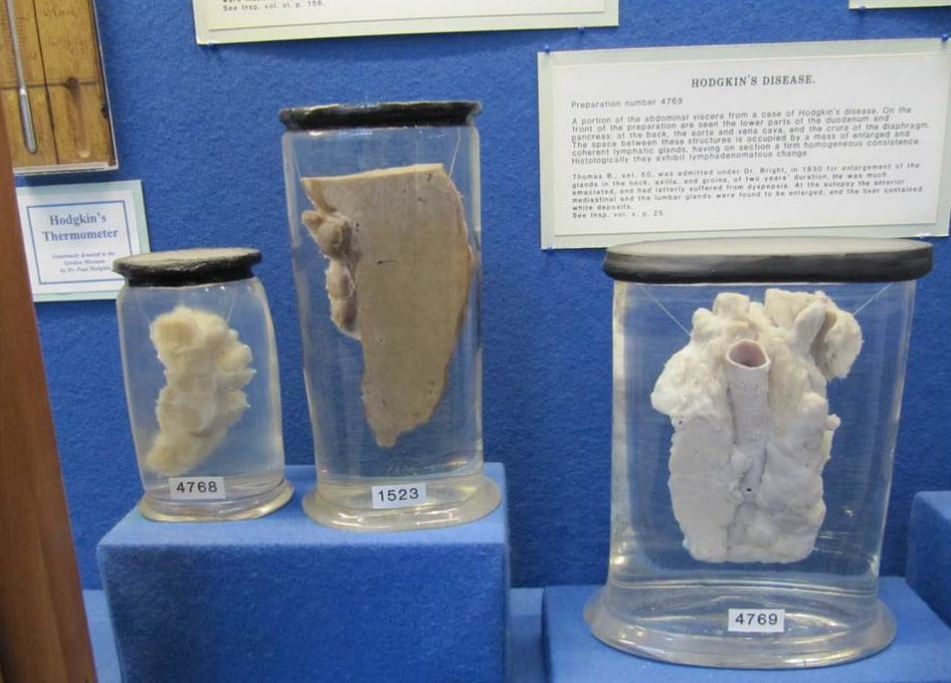
Three of the Original Post-Mortem Specimens of Organs Removed from Patients Who Succumbed to the Disease Currently Known as Hodgkin Lymphoma. The specimens are exhibited at Gordon Museum, King’s College, London, Guy’s Campus, Hodgkin House. Photo by E.J. Dann.

Thomas Hodgkin has made a vital contribution to the curriculum of medical education, a contribution that remains largely intact to this day. In 1827, he lectured at the opening of the academic year. His presentation entitled “On medical education” was a provocative talk on the superiority of the Continental way of studying medicine compared to the English system. The lecture was happily cited in *The Lancet*, which was very critical about the English health care system at that time. In the same year, Hodgkin introduced the course of morbid anatomy to Guy’s Hospital, a course that was initially optional, but which in time developed into the pathology course that became an integral and essential part of the curriculum at every medical school throughout the world. Hodgkin wrote a two-volume textbook, *On the Morbid Anatomy of the Serous and Mucous Membranes*. While the first part was published in 1836, when he was still at Guy’s, the second volume was issued only in 1840.

Thomas Hodgkin used his medical and organizational skills for the benefit of humanitarian missions around the world. He played a pivotal part in the foundation of the British Syrian Relief Fund initiated by Sir Moses Montefiore. Donations to the fund were not limited to private sources only; large contributions were also made by the governments of the United Kingdom, France, and Scandinavian countries. The fund-raising committee supplied medical aid to the survivors of the massacre that happened in Syria and Lebanon following ethnic riots in 1860. The committee used the remaining money to establish asylums for widows and orphans. This gives another proof that Hodgkin’s deeds are relevant today when the world is facing a humanitarian crisis, with millions of refugees fleeing persecution and violence.

Thomas Hodgkin died in Jaffa on April 4, 1866. He was buried in the Anglican cemetery, and his friend Montefiore shipped a red granite obelisk stone to be placed on his tomb (Figure 4). The inscription on the tombstone says that it was erected by Sir Moses Montefiore, Bart. “in commemoration of a friendship of more than 40 years and many journeys taken together in Europe, Asia and Africa.” The epitaph on the tomb is inscribed by Hodgkin’s deeply sorrowing widow and brother to record their irreparable loss “In the faith and hope of the gospel. *Humani nihil a se alienum putabat*” (Nothing of humanity was foreign to him). Hodgkin used a similar inscription on his thesis dedicated to the scientist whom he admired greatly, Alexander von Humboldt.

**Figure 4:**
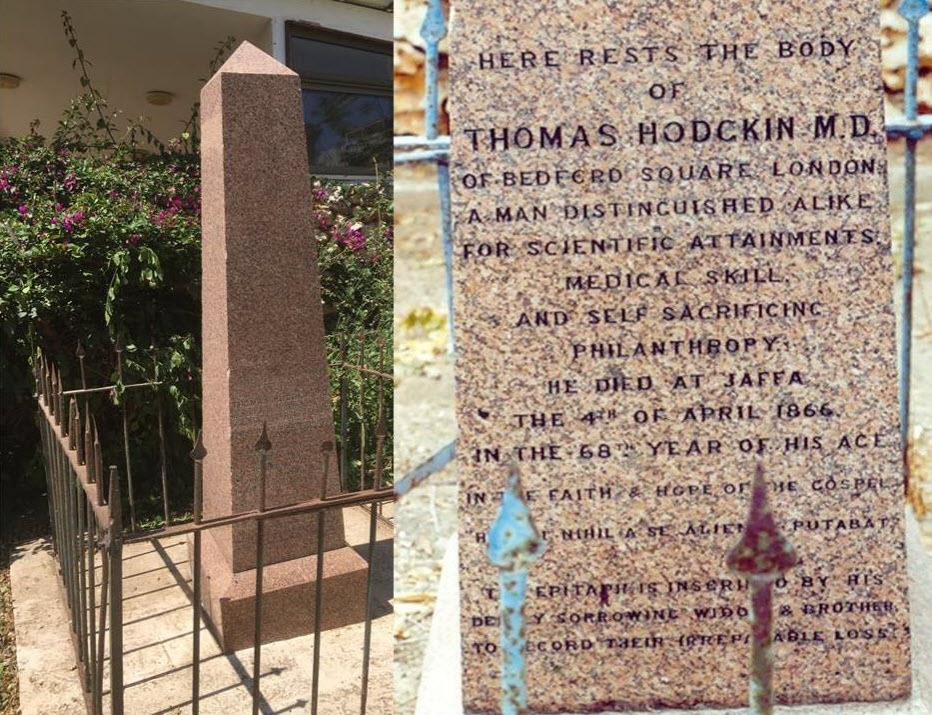
The Inscription on the Obelisk Stone Placed on Thomas Hodgkin’s Tomb. Photo by E.J. Dann.
